# Semi Lobar Holoprosencephaly with Vertebral Segmentation Defects

**Published:** 2017

**Authors:** Birendra RAI, Farhana SHARIF

**Affiliations:** 1 Department of Pediatrics, Midland Regional Hospital, Mullingar, Ireland; 2 Department of Pediatrics, Midland Regional Hospital, Mullingar, Ireland; 3 Royal College of Surgeons, Dublin, Ireland

**Keywords:** Holoprosencephaly, Vertebral segmentation defects, Brain malformation, Developmental delay

## Abstract

Holoprosencephaly is the most common embryonic brain defect. Foetuses who survive during intrauterine life are born with varying grades of brain and facial deformities. Extra craniofacial manifestations are common. Vertebral segmentation defects are rarely seen with holoprosencephaly, mainly in association with holoprosencephaly diencephalic hamartoblastoma (HDH) association. A female infant was born at term by normal delivery. Birth head circumference was below the 3rd percentile. Antenatal scan had showed microcephaly as the only abnormality. Physical examination revealed microcephaly, ocular hypotelorism, left ear skin tag and short neck. MRI of the brain showed semilobar holoprosencephaly. Neck radiograph revealed gross vertebral segmentation defect involving cervical and upper thoracic vertebrae.

She had initial feeding difficulties. She showed severe global developmental delay and had underlying central diabetes insipidus. Vertebral segmentation defect is rare in holoprosencephaly.

## Introduction

Holoprosencephaly is the most common developmental defect of the embryonic brain (1). Recent advancements in genomic knowledge base and powerful analytical methods have helped in identifying several underlying genetic aberrations related with holoprosencephaly but the search is still incomplete. 

Various authors have tried to fit the clinical phenotypes of holoprosencephaly into different syndromes. Diversity of clinical phenotype and its varying associations have not only made genotype phenotype correlation a difficult task but also have questioned the syndromic assignments of these varying phenotypes related with holoprosencephaly (2). Holoprosencephalic diencephalic hamartoblastoma (HDH) association is a clinical subset of holoprosencephaly, which has been associated with vertebral segmentation defects (3, 4).

Our case had very characteristics vertebral segmentation defects without any hamartomatous lesion in the brain.

## Case Report

A female infant was born at term by normal vaginal delivery to a primigravida mother in a regional hospital in Ireland. APGARs were reassuring at birth. Birth weight, length and head circumference were 3.2 kg (50^th^ percentile), 51 cm (50th percentile), and 30.5 cm (<3^rd^ percentile) respectively. Antenatal ultrasound scan at 28th week of gestation had revealed microcephaly.

The antenatal course was uneventful with preventive serology, normal screens, no alcohol or any other teratogenic drug use and no medical problems. After birth, our patient developed hypoglycaemia with sugar level of 1.9 mmol /L at the age of 6 h and was given intravenous bolus of 10% dextrose at 2 ml/kg. Her oral feeding was subsequently noticed to be poor requiring nasogastric supplementation.

Informed consent was taken from parents prior to publication of this report. No ethical approval was required.

Physical Examination revealed hypotelorism, left ear skin tag and short neck. A grade 3/6 systolic murmur was heard on cardiac examination. She had normal tone and reflexes and no neurological deficits were noted. 

Systemic examination and vital signs were within normal limits.

Cranial ultrasound performed for the evaluation of microcephaly and poor feeding could not visualize the midline separation plane of cerebral hemisphere. 

**Fig 1:**
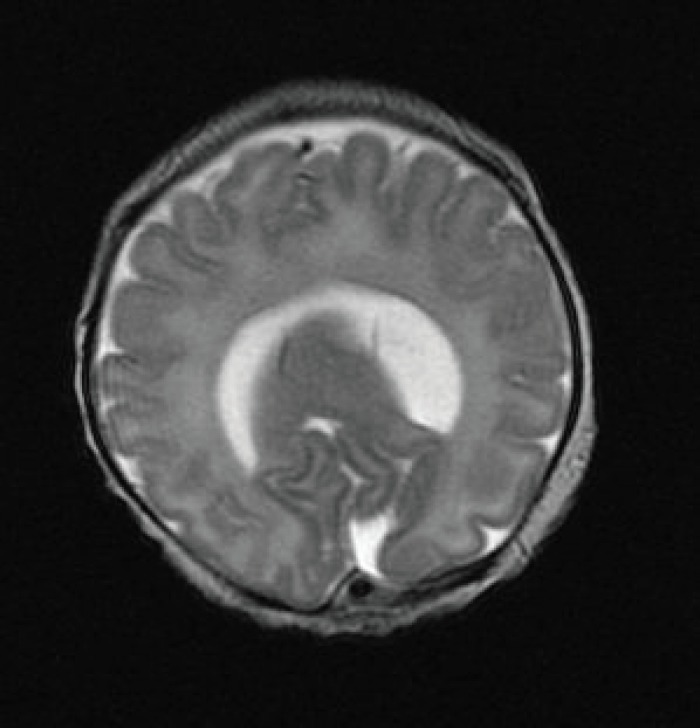
T2 weighted MRI of the brain showing horse shoe lateral ventricle with fused frontal lobe anteriorly

**Fig 2:**
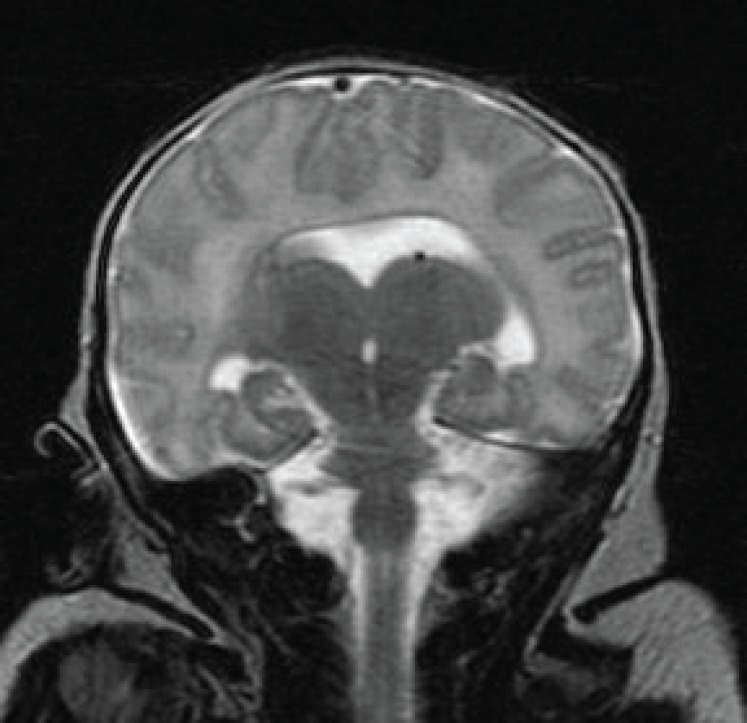
Coronal T2 weighted MRI of the brain showing fused frontal lobes of both the cerebral hemisphere

Magnetic resonance imaging of the brain showed a single horseshoe shaped lateral ventricle with temporal horns but no development of anterior horns. The frontal lobes were completely fused in the midline anteriorly but there was some interhemispheric fissure formation posteriorly (Figure 1 and 2). Thalami and caudate nuclei were partly fused and third ventricle was rudimentary. 

**Fig 3:**
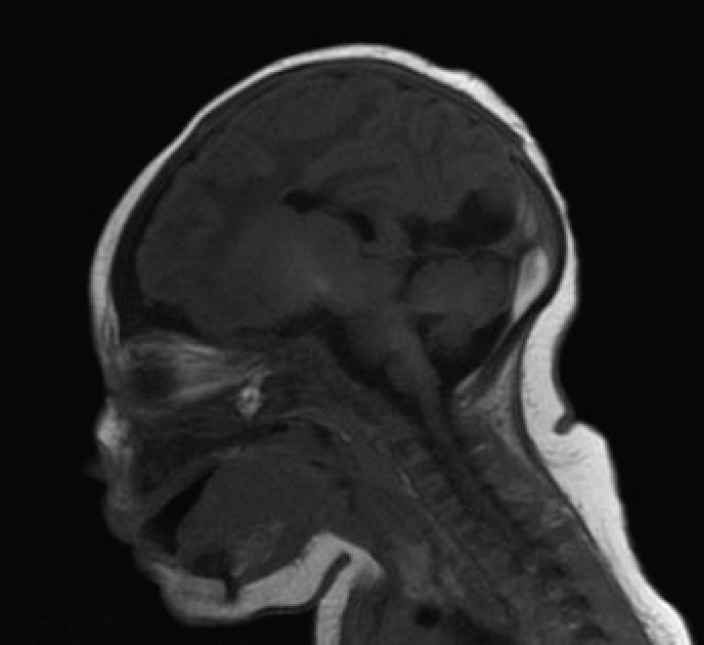
Sagittal T1 flair MRI image showing missing anterior part of the corpus callosum

**Fig 4:**
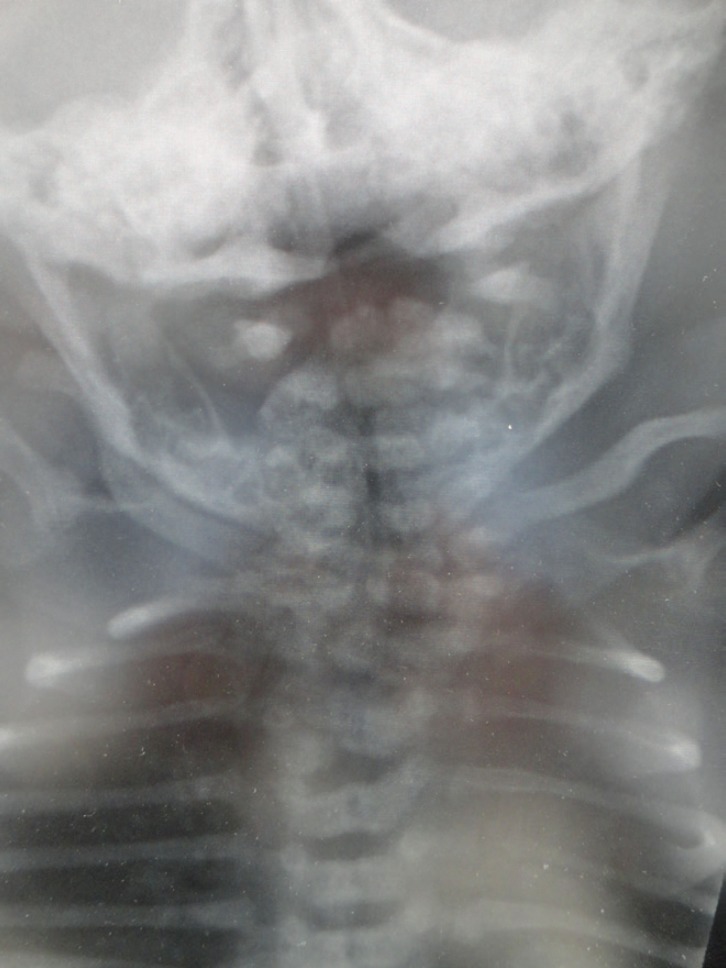
Neck radiograph showing vertebral segmentation defect involving cervical and upper thoracic vertebrae

Fourth ventricle was normally present. Anterior part of the corpus callosum was missing with normal presence of posterior part (Figure 3). Pituitary gland, cortex, white matter, cerebellum and brainstem were normal. These findings suggested semilobar holoprosencephaly. Neck radiograph revealed gross cervical and upper thoracic vertebral segmentation defect (Figure 4). Abdominal ultrasound was reportedly normal. Echocardiograph revealed a tiny patent ductus arteriosus with a 6 mm muscular ventricular septal defect. 

During her course in special care baby unit she developed hypernatremia with sodium 160 mmol/ L, chloride 114 mmol/ L, urea 3.4 mmol/ L and creatinine 50 mmol/ L.

Serum osmolality was 305 mili osmoles/ kg. Serum anti diuretic hormone level was 3 pmol/ L (3.7-11 pmol/ L).

She was supplemented with desmopressin for clinical impression of central diabetes insipidus and her urea and electrolytes were regularly monitored. Endocrine panel revealed normal luteinising hormone, thyroid function tests, cortisol, IGFBP3 and growth hormone levels. Metabolic Panel was normal. Cytogenetic study revealed normal female karyotype. Genetic mutation study including microarray could not identify any relevant genetic aberration of holoprosencephaly. 

Nasogastric nutrition was continued until full oral feeds were established. Later video fluoroscopy study showed aspiration of fluid on swallowing. Regular follow up in outpatient clinic revealed delay in all the areas of development with gross and fine motor being most affected. Both upper and lower limbs were hypertonic. She never had any convulsion. Her serum electrolytes and osmolality are well controlled on oral supplementation of desmopressin.

## Discussion

Holoprosencephaly is the most common defect of the developing embryonic brain due to disruption of ventral patterning process of the forebrain that is normally completed by the fifth week of gestation. Structural disturbance in the midline division of prosencephalon leads to varying degrees of brain and facial phenotypic anomalies. 1/250 early embryos are affected and most (more than 99 percent) of the affected embryos die in intrauterine period rendering an incidence of 1/10,000 to 1/20,000 live birth (1). The etiology of holoprosencephaly is multifactorial. Almost 40 to 50% of the cases are associated with chromosomal anomaly with trisomy 13 being most common (5). The rest are either part of well-known syndromes or unknown in cause. Most of the cases are sporadic. 

As per degree and region of neocortical nonseparation, holoprosencephaly has been divided into 4 subtypes (6). Most common and most severe, noted in about 45 % of the cases (5) is the alobar form in which cerebral hemisphere fails to divide along its entire length. 

Olfactory bulbs, tracts and corpus callosum remain absent. Cyclopia (single eye in midline), ethmocephaly, and cebocephaly are some of the prominent external facial features noted in alobar form. Semilobar form is the next bigger group in which interhemispheric fissure divides the posterior part into two halves but frontal and parietal cortex are still conjoined together with varying absence of deep gray matter nuclei. Olfactory bulbs, tracts and corpus callosum may or may not be absent. 

Ocular hypotelorism, flat facies, and midline clefting of face are some of the prominent features, which remain present in varying severity. This form is compatible with life.

In a continuous fashion, interhemispheric fissure bifurcates lateral ventricle completely but fails to separate frontal cortex. This form is known as lobar form. Phenotypically this is very mild form. Here cerebral cortex is fully formed along with deep gray matter nuclei. Corpus callosum may be normal, hypoplastic or absent. The mildest end of the anatomic spectrum is midline interhemispheric form in which posterior frontal cortex and part of parietal cortex fails to bifurcate. Lateral ventricles are completely separated. 

Extra craniofacial manifestations of holoprosencephaly are genital anomalies (24%), post axial polydactyly (8%), vertebral defects (5%), limb reduction defects (4%) and cardiac anomaly mainly transposition of great arteries (4%) (7). More than 20 syndromes have so far been reported with holoprosencephaly due to varying combinations of different phenotypic features in different case series of holoprosencephaly. Most notables are Pallister Hall syndrome due to gene GLI3 mutation and Smith Lemli Opitz syndrome due to gene DHCR7 mutation. Verlos et al. (2) questioned the separate existence of Pallister Hall syndrome citing it as extremes of spectrum of one or other syndrome. They suggested creation of concept of multiplex phenotype, “Cerebro-Acro-Visceral Early lethality multiplex syndrome” to encompass all the ambiguous cases. Guimiot et al. (3) in the study of their 5 foetuses with diencephalic hamartoma could not detect GLI3 mutation which is seen in Pallister Hall syndrome with diencephalic hamartoma. They noted holoprosencephaly, cardiac anomaly and vertebral anomaly in the cohort of their patients. Castori et al. (4) noted vertebral segmentation defect in 50 % of their cohorts of Holoprosencephaly-Diencephalic Hamartoblastoma (HDH) association. Our patient had characteristics vertebral segmentation defects of upper cervical and thorasic vertebrae, ventricular septal defect and left ear skin tag along with Holoprosencephaly but had no evidence of hamartoblastoma in diencephalon. 

In accordance with the grades of holoprosencephaly and severity of CNS malformations, multiple neurodevelopmental and endocrine problems are encountered in surviving patients (8). Feeding difficulties along with delays in gross and fine motor skills are universal. Language and cognitive delay can be present to varying degree. Semilobar, Lobar and Midline Interhemispheric forms may attain adulthood with varying disability (9). Central Diabetes Insipidus is the most common endocrine manifestation. Midline Interhemispheric forms have been shown to have no endocrine dysfunction in one study (10). Respiratory and cardiac morbidity, repeated pulmonary infections, diabetes insipidus and seizure disorders are some of the common conditions leading to death (9). Vertebral segmentation defects may exacerbate pulmonary morbidity.

Several genetic mutations have been identified to date to cause Holoprosencephaly. Sonic hedge hedge (SHH), ZIC2, SIX3, TGIF, PATCHED1, TDGF1/ CRIPTO, FAST1, GLI2,GLI3, and DHCR are some of the well-known genes involved in pathogenesis of holoprosencephaly (11, 12). Many more are under investigation. Other factors reported to be involved in pathogenesis of holoprosencephaly are maternal diabetes, retinoic acid, ethyl alcohol, aspirin use and reduced maternal to either foetal cholesterol transport or defective cholesterol biosynthesis in foetus itself (13, 14).

Meticulous scanning technique can help with antenatal diagnosis (15). Management involves multidisciplinary team approach.

**In Conclusion, **holoprosencephaly and its wide spectrum syndromic association still poses challenges to researchers; with its complex associations making it sometime difficult to fit any particular patient into a proper subtype. Furthermore, vertebral segmentation defects, which are widely reported to be associated with holoprosencephaly with diencephalic hamartoblastoma (HDH) association, can rarely be found with holoprosencephaly only, as described in this case report. 

## Author’s Contribution

Rai B: Literature Review and Manuscript drafting

Sharif F: Clinical diagnosis, Patient management. Critical revision of the intellectual content of the manuscript.

All authors agreed to be accountable for all aspects of the work in ensuring that questions related to the accuracy or integrity of any part of the work are appropriately investigated and resolved.

## Conflict of Interests:

None

## References

[1] Shiota K, Yamada S, Komada M (2007). Embryogenesis of holoprosencephaly. Am J Med Genet A.

[2] Verloes A, Gillerot Y, Langhendries JP (1992 ). Variability versus heterogeneity in syndromal hypothalamic hamartoblastoma and related disorders: review and delineation of the cerebro-acro-visceral early lethality (CAVE) multiplex syndrome. Am J Med Genet.

[3] Guimiot F, Marcorelles P, Aboura A (2009). Giant diencephalic harmartoma and related anomalies: a newly recognized entity distinct from the Pallister-Hall syndrome. Am J Med Genet A.

[4] Castori M, Douzgou S, Silvestri E (2007). Reassessment of holoprosencephaly-diencephalic hamartoblastoma (HDH) association. Am J Med Genet A.

[5] Croen LA, Shaw GM, Lammer EJ (1996). Holoprosencephaly: epidemiologic and clinical characteristics of a California population. Am J Med Genet.

[6] Christèle Dubourg, Claude Bendavid, Laurent Pasquier (2007). Holoprosencephaly. Orphanet J Rare Dis.

[7] Orioli IM, Castilla EE (2010). Epidemiology of holoprosencephaly: Prevalence and risk factors. Am J Med Genet C Semin Med Genet.

[8] Plawner LL, Delgado MR, Miller VS (2002). Neuroanatomy of holoprosencephaly as predictor of function: beyond the face predicting the brain. Neurology.

[9] Barr M Jr, Cohen MM Jr (1999). Holoprosencephaly survival and performance. Am J Med Genet.

[10] Lewis AJ, Simon EM, Barkovich AJ (2002). Middle interhemispheric variant of holoprosencephaly: a distinct cliniconeuroradiologic subtype. Neurology.

[11] Bellone S, De Rienzo F, Prodam F (2010). Etiopathogenetic advances and management of holoprosencephaly: from bench to bedside. Panminerva Med.

[12] Wallis D, Muenke M (2000). Mutations in holoprosencephaly. Hum Mutat.

[13] Miller EA, Rasmussen SA, Siega-Riz AM (2010). National Birth Defects Prevention Study Risk factors for nonsyndromic holoprosencephaly in the National Birth Defects Prevention Study. Am J Med Genet C Semin Med Genet.

[14] Cohen MM Jr, Shiota K (2002). Teratogenesis of holoprosencephaly. Am J Med Genet.

[15] Kline-Fath BM, Calvo-Garcia MA (2011). Prenatal imaging of congenital malformations of the brain. Semin Ultrasound CT MR.

